# Loss of Cathepsin B and L Leads to Lysosomal Dysfunction, NPC-Like Cholesterol Sequestration and Accumulation of the Key Alzheimer's Proteins

**DOI:** 10.1371/journal.pone.0167428

**Published:** 2016-11-30

**Authors:** Stjepko Cermak, Marko Kosicek, Aleksandra Mladenovic-Djordjevic, Kosara Smiljanic, Selma Kanazir, Silva Hecimovic

**Affiliations:** 1 Division of Molecular Medicine, Rudjer Boskovic Institute, Croatia; 2 Laboratory of Molecular Neurobiology, Department of Neurobiology, Institute for Biological Research ''Sinisa Stankovic'', University of Belgrade, Belgrade, Serbia; Torrey Pines Institute for Molecular Studies, UNITED STATES

## Abstract

Proper function of lysosomes is particularly important in neurons, as they cannot dilute accumulated toxic molecules and aggregates by cell division. Thus, impairment of lysosomal function plays an important role in neuronal degeneration and in the pathogenesis of numerous neurodegenerative diseases. In this work we analyzed how inhibition and/or loss of the major lysosomal proteases, the cysteine cathepsins B and L (CtsB/L), affects lysosomal function, cholesterol metabolism and degradation of the key Alzheimer's disease (AD) proteins. Here, we show that cysteine CtsB/L, and not the aspartyl cathepsin D (CtsD), represent a major lysosomal protease(s) that control lysosomal function, intracellular cholesterol trafficking and AD-like amyloidogenic features. Intriguingly, accumulation of free cholesterol in late endosomes/lysosomes upon CtsB/L inhibition resembled a phenotype characteristic for the rare neurodegenerative disorder Niemann-Pick type C (NPC). CtsB/L inhibition and not the inhibition of CtsD led to lysosomal impairment assessed by decreased degradation of EGF receptor, enhanced LysoTracker staining and accumulation of several lysosomal proteins LC3II, NPC1 and NPC2. By measuring the levels of NPC1 and ABCA1, the two major cholesterol efflux proteins, we showed that CtsB/L inhibition or genetic depletion caused accumulation of the NPC1 in lysosomes and downregulation of ABCA1 protein levels and its expression. Furthermore, we revealed that CtsB/L are involved in degradation of the key Alzheimer’s proteins: amyloid-β peptides (Aβ) and C-terminal fragments of the amyloid precursor protein (APP) and in degradation of β-secretase (BACE1). Our results imply CtsB/L as major regulators of lysosomal function and demonstrate that CtsB/L may play an important role in intracellular cholesterol trafficking and in degradation of the key AD proteins. Our findings implicate that enhancing the activity or levels of CtsB/L could provide a promising and a common strategy for maintaining lysosomal function and for preventing and/or treating neurodegenerative diseases.

## Introduction

Lysosomes are cellular organelles with a crucial role in the degradation of macromolecules. In addition to representing end points of autophagic, endocytic and phagocytic pathways, recent studies have demonstrated their role in a wide range of biological processes such as plasma membrane repair and the immune response [1]. They are filled with more than 60 different acid hydrolases that produce lysosomal catabolites which are then transported out of lysosomes via specific transporters or via vesicular membrane trafficking for energy homeostasis or molecular biosynthesis [2]. Among these the term cathepsin refers to serine proteases cathepsins A and G, aspartic proteases cathepsins D and E, and cysteine proteases cathepsins B, C, L, F, H, K, O, S, V, X and W.

The idea that cathepsins play an important role in the pathogenesis of neurodegenerative disorders has been long known in the scientific literature. Changes in cathepsin concentration, activity and localization are normally found in aging neurons and are considered as a cause of age-related neuropathologic changes [3]. Cathepsins B (CtsB) and D (CtsD) have been found at extracellular sites closely associated to senile plaques in patients suffering from Alzheimer's disease (AD) [4]. Some authors have demonstrated that cathepsins B and L (CtsB and CtsL) could even possess a β-secretase activity in the production of amyloid-β (Aβ) peptides [5–8]. Furthermore, inhibition of CtsB and CtsL has also shown beneficial effects in reduction of Aβ oligomerization and plaque formation [9]. Mouse models of AD treated with small doses of CtsB/L inhibitors have demonstrated a reduction in Aβ peptide levels and an improvement of synaptic and behavioral deficits accompanied by increased CtsB levels [10]. Increased CtsB and CtsD levels have also been found in Niemann-Pick type C disease [11], a rare, inherited, lysosomal storage disease that shares many pathological hallmarks with AD [12]. In contrast, genetic ablation of CtsB in AD mice resulted in increased amounts of Aβ1–42 and a more abundant plaque deposition pattern, suggesting an antiamyloidogenic role of CtsB [13]. Mice that lack both CtsB and CtsL (CtsB^-/-^L^-/-^) have shown extensive neurodegeneration and pronounced reactive astrocytosis [14], main hallmarks of both AD and NPC. Perturbation of cholesterol metabolism, another phenotypical characteristic of both AD an NPC, has been reported in macrophages treated with a CtsB/L inhibitor [15].

The objective of this study was to elucidate the link between lysosomal dysfunction, cholesterol homeostasis and the production of amyloidogenic products of the APP protein cleavage–a key event in the pathogenesis of AD. We have hypothesized that inhibition of cysteine proteases, CtsB and CtsL, causes a specific lysosomal dysfunction that leads to dysfunction of intracellular cholesterol transporters, changes in vesicular trafficking of the key AD proteins leading to an increased amyloidogenic cleavage of the APP protein. Our findings indicate that the activity of cysteine proteases CtsB and CtsL is interconnected with the intracellular cholesterol accumulation and the development of AD-like amyloidogenic features. Thus, keeping the lysosomes functional is important for both preventing and treating this devastating disorder.

## Materials and Methods

### Antibodies

ABCA1 (rabbit polyclonal, Novus Biologicals, RRID: AB_10000630, WB 1:500); β-actin (mouse monoclonal, Cell Signaling, RRID: AB_2242334, WB 1:10000); APP N-terminal [22C11] (mouse monoclonal, Millipore, RRID: AB_94882, WB 1:1000); APP C-terminal [2C11] (mouse polyclonal, supernatant from hybridoma cells, provided by Dr. Stefan Lichtenthaler, German Center for Neurodegenerative Diseases, Germany, WB 1:10); APP C-terminal [6687] (rabbit polyclonal, provided by Dr. Christian Haass, German Center for Neurodegenerative Diseases, Germany, IP 1:300); BACE1 (rabbit monoclonal, Cell Signaling, RRID: AB_1903900, WB 1:1000); Cathepsin B (rabbit polyclonal, Abcam, RRID: AB_725974, WB 1:500); EGFR (rabbit monoclonal, Cell Signaling, RRID: AB_10828841, WB 1:1000); LAMP1 (rabbit polyclonal, Sigma-Aldrich, RRID: AB_477157, WB 1:1000); LC3 (rabbit polyclonal, Sigma-Aldrich, RRID: AB_1079382, WB 1:500); NPC1 [EPR5209] (rabbit monoclonal, Abcam, Cat# ab134113, WB 1:1000, ICC 1:100); NPC2 [HL5895] (rabbit polyclonal, provided by Dr. Peter Lobel, Center for Advanced Biotechnology and Medicine, New Jersey, USA, WB 1:1000); α-tubulin [DM1A] (mouse monoclonal, Cell Signaling, Cat# 3873, WB 1:2000).

### Cell lines

Chinese hamster ovary cells (CHOwt) and CHO *NPC1*-null cells (kindly provided by Dr. Daniel S. Ory, Washington University School of Medicine, USA) were maintained in DMEM/F12 medium (1:1) supplemented with 10% Fetal Bovine Serum, 2 mM L-glutamine and antibiotic/antimycotic mix (all from Sigma-Aldrich) [16].

Human neuroblastoma cells (SH-SY5Y) (kindly provided by Dr. Stefan Lichtenthaler, German Center for Neurodegenerative Diseases, Germany) were maintained in DMEM/F12 medium (1:1) supplemented with 15% Fetal Bovine Serum, 2 mM L-glutamine, antibiotic/antimycotic mix (all from Sigma-Aldrich) and 1% non-essential amino acids (Gibco) [17].

Wild type mouse embryonic fibroblasts (MEFwt), cathepsin B/L knockout (KO) MEFs (CtsB^-/-^, CtsL^-/-^, CtsB^-/-^L^-/-^) (kindly provided by Dr. Oliver Schilling, Institute for Molecular Medicine and Cell Research, University of Freiburg, Germany) and cathepsin D KO MEFs (CtsD^-/-^) (kindly provided by Dr. Paul Saftig, Institute of Biochemistry, Kiel, Germany) were maintained in DMEM GlutaMAX medium (Gibco) supplemented with 10% Fetal Bovine Serum and antibiotic/antimycotic mix (all from Sigma-Aldrich) [18;19].

### Pharmacological treatments

To mimic NPC phenotype CHOwt / SH-SY5Y cells were treated 24 h / 48 h with 2 μg/mL / 3 μg/mL U18666A (Sigma-Aldrich) in growth medium, respectively. For cathepsin B/L inhibition CHOwt and SH-SY5Y cells were treated 24 h with 100 μM PADK (Bachem, Switzerland) in growth medium. For cathepsin D inhibition CHOwt and SH-SY5Y cells were treated 24 h with 10 μg/mL Pepstatin A (Sigma-Aldrich) in growth medium. To block lysosomal activity CHOwt and SH-SY5Y cells were treated 24 h with 100 μM Leupeptin (Sigma-Aldrich) and 5 mM NH_4_Cl (Kemika, Croatia) in growth medium.

### EGFR degradation assay

SH-SY5Y cells were grown in 6 well plates and treated as described in *Pharmacological treatments* section. The cells were then incubated with 100 ng/mL human epidermal growth factor (EGF, Sigma-Aldrich) for 1, 2 or 3 hours. The cells were then collected and processed as described in *Western blot* section.

### Western blot

The cells were washed with PBS and collected in lysis buffer (50mM Tris·HCl, pH 7.6, 150mM NaCl, 2mM EDTA, 1% NP40, 0,5% Triton X-100) supplemented with protease inhibitor cocktail (Roche Applied Science, Germany). After incubation on ice for 30 min the lysates were centrifuged at 4°C for 10 min at 16000 x g. Supernatants were collected and total protein concentration was measured using commercially available Pierce BCA Protein Assay Kit (Thermo Scientific, USA) according to manufacturer’s protocol. Supernatants were mixed with 6x sample buffer (60% glycerol, 12% SDS, 3% DTT, 1/8 v/v 0.5 M Tris pH 6.8, bromphenol bleu) and heated at 70°C for 10 min. The same amounts of proteins were loaded to Tris-Glycine gels.

Endogenous APP-CTF levels were detected by immunoprecipitation using APP C-terminal antibody 6687 and Protein A Sepharose (Sigma-Aldrich) at 4°C overnight with constant rocking. The immunoprecipitates were washed twice with lysis buffer and once with PBS buffer. Samples were heated at 70°C for 10 min in 2x sample buffer and loaded to Tris-Tricine gel.

After SDS-PAGE proteins were transferred to PVDF membrane (Roche Applied Science, Germany), and subjected to blotting using HRP conjugated secondary antibodies (Bio-Rad). Proteins were visualized by chemiluminescence using POD chemiluminescence blotting substrate (Roche Applied Science, Germany). β-Actin was used as a loading control for CHO cells, while α-tubulin was used as a loading control for SH-SY5Y and MEF cells. Western blots were quantified using ImageJ software (National Institutes of Health, USA). Statistical validation of the data was achieved by Student t-test.

### ELISA Amyloid-β

For Aβ40 measurement, cells were grown in one Ø10 cm-plate and were transiently transfected with APPwt-6myc plasmid vector using Lipofectamine LTX (Invitrogen, USA), according to the supplier's instructions. Forty-eight hours after transfection cells were washed and collected in PBS. Cells were spin at 4°C for 5 min at 1,000 × g and cell pellet was resuspended and lysed in either 500 μL RIPA buffer containing a protease inhibitor cocktail (Roche, Germany) for analysis of soluble (RIPA-extractable) intracellular Aβ40 or were resuspended and lysed in 100 μL of formic acid (Fluka, Germany) per plate for analysis of insoluble (FA-extractable) intracellular Aβ40. Lysates were incubated 10 min on ice and were passed 10 times through 23-G needle and centrifuged at 4°C for 30 min at 16,000 × g. After centrifugation FA supernatants were neutralized with 1.9 mL of 1M Tris. RIPA-lysates were used immediately for analysis of intracellular Aβ40 and for measuring protein concentration. The medium was collected and used for analysis of secreted Aβ40. The levels of secreted and intracellular Aβ40 were determined by ELISA Aβ40 kit (Invitrogen, USA) according the manufacturer protocol. Absorption was measured at 450 nm (Multiskan EX, Thermo Scientific, USA). Every sample was measured in technical duplicates and average value was used as a relevant value. Aβ40 levels were normalized to total protein concentration in the sample. Statistical validation of the data was achieved by Student t-test using Excel software (Microsoft, USA).

### Confocal microscopy

The cells were grown on Ø12 mm-coverslips and treated as described in *Pharmacological treatments* section. All samples were analyzed using inverted fluorescent confocal microscope (Leica SP8 X FLIM).

Accumulation of intracellular free cholesterol was monitored using filipin (Sigma-Aldrich, USA). Cells grown on coverslips were rinsed twice in PBS, following fixation in 4% paraformaldehyde (PFA, Sigma-Aldrich, USA) in PBS. The cells were rinsed in PBS again and incubated with 100 μg/mL filipin working solution for 1 h in the dark at room temperature. After washing the cells twice in PBS and once in deionized water, the cells were mounted (Polyvinyl alcohol mounting medium with DABCO antifading, Fluka, Switzerland).

Lysosomal dysfunction was monitored using LysoTracker Red DND-99 (Molecular Probes, USA). The cells were treated for 80 min with 200 nM LysoTracker in growth medium, washed with PBS, fixed in 4% PFA and stained with 0.5% Hoechst (ImmunoChemistry Technologies, USA) in PBS for 30 min. After washing the cells twice in PBS and once in deionized water, the cells were mounted.

For NPC1 immunostaining, cells grown on coverslips were rinsed twice in PBS, fixed in 4% PFA, permeabilized with 0.2% saponin (Sigma-Aldrich) and blocked in 5% goat serum (Sigma-Aldrich). Immunostaining was performed with primary antibody at 4°C overnight in a dark wet chamber, following incubation with secondary Alexa488 conjugated antibody (Invitrogen). After washing the cells twice in PBS and once in deionized water, the cells were mounted.

For APP and BACE1 colocalization experiment cells grown on coverslips were transiently transfected with BACE1-GFP and APP-RFP plasmid vector (obtained from Dr. Bjorn von Einem, Ulm University, Germany) using Lipofectamine LTX (Invitrogen, USA), according to the supplier's instructions. 48 h after transfection, the cells were washed, fixed and mounted as described above.

### Real time PCR

Total RNA was prepared from treated and untreated SH-SY5Y cells in three independent experiments using Trizol reagent (Thermo Fisher Scientific) according to the manufacturer’s instructions. RNA integrity was checked by electrophoresis. RNA was treated with 1 U/μg RNA of RNase-free DNase I (Thermo Fisher Scientific) and reverse transcribed (RT) into cDNA with random hexamer primers using a High-Capacity cDNA Reverse Transcription Kit (Applied Biosystems) under RNase-free conditions, at 25°C for 10 min, 37°C for 2 h and 85°C for 5 minutes. The cDNA was stored at -20°C until use. Real Time RT- PCR reactions were performed by the TaqMan gene expression assay (Applied Biosystems) using Assays-on-Demand Gene Expression product for β-actin (assay ID Hs99999903_m1), GAPDH (assay ID Hs03929097_g1), 18sRNA (Assay ID Hs99999901_s1), ABCA1 (assay ID Hs01059118_m1), and NPC1 (assay ID Hs00264835_m1). The reactions were performed in a 25 μL reaction mixture containing 1x TaqMan Universal Master Mix with AmpErase UNG, 1x Assay Mix (Applied Biosystems) and cDNA template (1 ng of RNA converted to cDNA). PCR reactions were performed in the ABI prism 7000 Sequence Detection System at 50°C for 2 min, 95°C for 10 min, followed by 40 cycles at 95°C for 15 s, and at 60°C for 1 min. The experimental threshold was calculated from the mean baseline fluorescence signal from cycles 3 to 15, plus 10 standard deviations. The threshold cycle (Ct) was defined for each amplification plot. Each sample was run in triplicate and mean Ct values were used for further calculations.

According to the validation experiment, we detected that for all three examined genes (actin, GAPDH and 18sRNA), expression was not regulated or influenced by the experimental treatments, and thus all of them could be used as housekeeping genes–HKG (data not shown). We selected actin as a suitable internal standard for further quantification studies of cholesterol egress gene expression under the treatments and included in every analysis to correct for differences in inter-assay amplification efficiency. Quantification was performed by the 2-DDCt method. The obtained results were tested for statistical significance (P ˂ 0.05) using RQ Study Add ON v 1.1 software (Applied Biosystems). The fold-changes of the mRNA levels of all samples were expressed relative to the calibrator, untreated sample that served as a control (100%).

## Results

### Inhibition of cathepsin B/L and not cathepsin D causes lysosomal dysfunction

**Fig 1:**
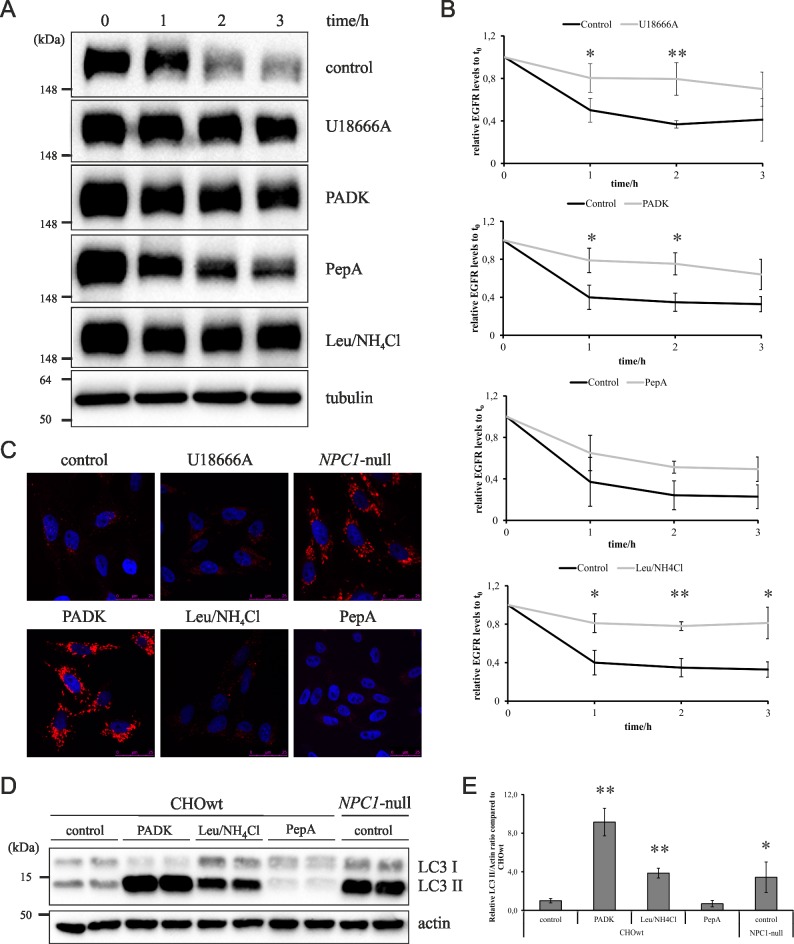
Inhibition of cathepsin B/L and not cathepsin D causes lysosomal dysfunction. (A) EGFR degradation assay. Western blot analysis of EGFR in SH-SY5Y cells treated with different inhibitors at different time points. α-Tubulin was used as a loading control. (B) Quantification of Western blot results of the 3 independent experiments was performed by ImageJ. Student t-test was used for statistical analysis. Error bars present the mean ± standard deviation (* p < 0.05, ** p < 0.01). (C) Confocal microscopy of CHOwt cells treated with different inhibitors and CHO *NPC1*-null cells. LysoTracker (red) and Hoechst (blue). (D) Western blot of CHOwt cells treated with different inhibitors and CHO *NPC1*-null cells using LC3 antibody. β-Actin was used as a loading control. (E) Quantification of Western blot results of the 3 independent experiments was performed by ImageJ. Student t-test was used for statistical analysis. Error bars present the mean ± standard deviation (* p < 0.05, ** p < 0.01).

We have previously demonstrated that a lysosomal storage disorder Niemann-Pick type C (NPC), in which free cholesterol accumulates in late endosomes/lysosomes due to NPC1/NPC2 dysfunction, shows cholesterol-dependent accumulation of intracellular Alzheimer's Aβ and APP-CTF fragments [20]. To further investigate the link between lysosomal dysfunction, altered cholesterol metabolism and accumulation of Aβ/APP-CTFs we treated CHO cells and human neuroblastoma SH-SY5Y cells with a general lysosomal inhibitor Leupeptin and ammonium chloride (Leu/NH_4_Cl), cathepsin B/L inhibitor (PADK) or cathepsin D inhibitor Pepstatin A (PepA). In addition, we treated cells with U18666A compound that has previously shown to cause intracellular cholesterol accumulation and lysosomal dysfunction as seen in NPC disease or we used CHO cells in which *NPC1* gene has been deleted (CHO *NPC1*-null). The results of monitoring degradation of the EGF receptor (EGFR) as a measure of lysosomal degradation capacity in Fig 1 show that all treatments, except CtsD inhibition (PepA), caused significantly decreased lysosomal degradation of EGFR indicating lysosomal dysfunction (Fig 1A and 1B). This analysis was performed in human neuroblastoma SH-SY5Y cells since human EGF ligand that we used for monitoring EGFR uptake and its lysosomal degradation could not be used in CHO cells. Furthermore, labelling of the acidic lysosomal organelles in CHO cells with LysoTracker revealed the strongest LysoTracker signal in PADK-treated cells which was comparable to the LysoTracker signal in CHO *NPC1*-null cells (Fig 1C), supporting lysosomal impairment. Similarly, CtsB/L inhibition by PADK-treatment in CHOwt cells caused accumulation of LC3-II, a marker of autophagy activation and lysosomal degradation, and an increased ratio of LC3-II/actin as in Leu/NH_4_Cl-treated CHOwt cells and in CHO *NPC1*-null cells (Fig 1D and 1E). To analyze whether increased LC3II levels upon PADK- and Leu/NH_4_Cl-treatment are due to activation of autophagy and/or decreased lysosomal degradation we co-treated PADK and Leu/NH_4_Cl-treated cells as well as control untreated CHOwt-cells with rapamycin–a well known activator of autophagy. As shown in S1 Fig rapamycin treatment (+Rap) caused significant upregulation of LC3II/actin ratio in control CHOwt cells which was lower to the ratio obtained upon either PADK- or Leu/NH_4_Cl-treatment. In addition, co-treatment with rapamycin of PADK- and Leu/NH_4_Cl-treated cells caused a further increase in LC3II/actin ratio, which was comparable as in rapamycin-treated CHOwt cells, suggesting that the accumulated LC3II levels upon PADK- and Leu/NH_4_Cl-treatments are likely due to decreased degradation of LC3II and not the activation of autophagy.

### Cathepsin B/L inhibition or genetic depletion causes NPC disease-like cholesterol accumulation

**Fig 2:**
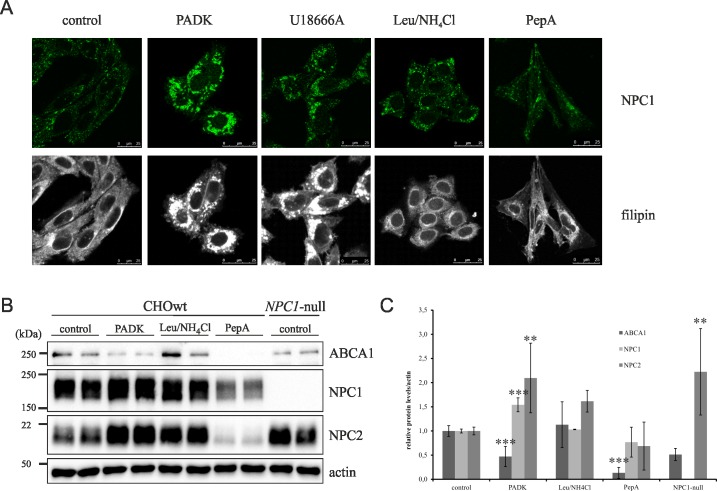
Cathepsin B/L inhibition causes NPC disease-like cholesterol accumulation in CHO cells. (A) Confocal microscopy of CHOwt cells treated with different inhibitors. Cholesterol (filipin staining, white) and NPC1 (green). (B) Western blot of CHOwt cells treated with different inhibitors using ABCA1, NPC1 and NPC2 antibody. β-Actin was used as a loading control. (C) Quantification of Western blot results of the 3 independent experiments was performed by ImageJ. Student t-test was used for statistical analysis. Error bars present the mean ± standard deviation (** p < 0.01, *** p < 0.001).

**Fig 3:**
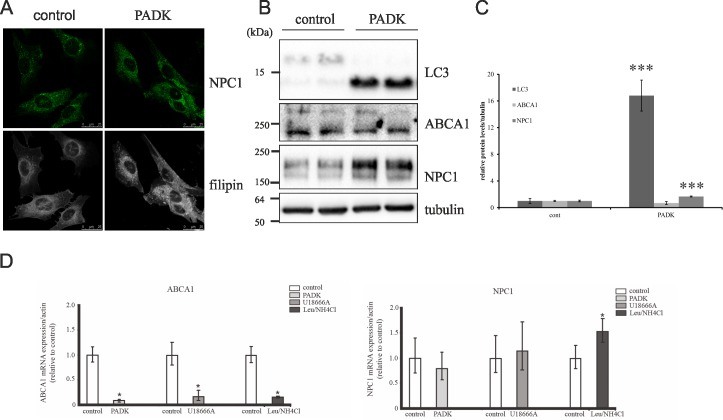
Cathepsin B/L inhibition causes NPC disease-like cholesterol accumulation in SH-SY5Y cells. (A) Confocal microscopy of SH-SY5Y control and PADK treated cells. Cholesterol (filipin staining, white) and NPC1 (green). (B) Western blot of SH-SY5Y control and PADK treated cells using LC3, ABCA1 and NPC1 antibody. α-Tubulin was used as a loading control. (C) Quantification of Western blot results of the 3 independent experiments was performed by ImageJ. Student t-test was used for statistical analysis. Error bars present the mean ± standard deviation (*** p < 0.001). (D) RT-PCR Expression of the cholesterol egress genes in the control, PADK, U18666A- and Leu/NH_4_Cl-treated SH-SY5Y cells, normalized to β-actin and quantified by 2−ΔΔCt method using control sample as calibrator. ATP-Binding Cassette sub-family A member 1 (ABCA1) and Niemann-Pick C1 protein (NPC1) mRNA levels presented as a fold change. The error bars present the mean ± SEM (* p < 0.05).

Our results of LysoTracker staining have indicated that blocking the activity of cathepsin B/L by PADK causes NPC-disease like lysosomal impairment (Fig 1C). To further investigate this we analyzed the accumulation of intracellular free cholesterol (measured by filipin staining), characteristic feature of NPC disease phenotype in PADK-treated cells as well as in cells treated with U18666A compound, Leupeptin/NH_4_Cl and Pepstatin A compared to untreated CHOwt cells (Fig 2A). In addition, we analyzed NPC1 immunostaining under the same conditions. Our results show that, in contrast to all other treatments (Leu/NH_4_Cl, PepA), PADK-treatment of CHOwt cells caused accumulation of intracellular free cholesterol similar as in U18666A treated cells (Fig 2A). In parallel, increased accumulation of NPC1 in enlarged lysosomal compartments was observed by immunostaining in both PADK and U18666A treated cells (Fig 2A). This increase was further confirmed by immunoblotting which showed increased levels of both NPC1 and NPC2, the two lysosomal cholesterol egress proteins, upon PADK treatment (Fig 2B and 2C). The NPC2, but not the NPC1, increase was also observed in Leu/NH_4_Cl-treated cells (although did not reach statistical significance). Interestingly, in contrast to PADK- and Leu/NH_4_Cl-treated cells, NPC1 and NPC2 levels were even slightly decreased (not statistically significant) in CtsD inhibitor-treated cells (PepA) (Fig 2B and 2C). The NPC disease-like cholesterol trafficking defect upon CtsB/L inhibition (PADK treatment) was further confirmed by analyzing the levels of cholesterol egress protein ABCA1, which were shown to be downregulated both in PADK treated CHOwt cells and in CHO *NPC1*-null cells (Fig 2B and 2C). The observed changes were also analyzed in human neuroblastoma SH-SY5Y cells which showed increased filipin staining upon PADK-treatment (Fig 3A) and statistically significant increase of LC3II and NPC1 levels (Fig 3B and 3C). The levels of ABCA1 protein in PADK-treated SH-SY5Y cells were only slightly decreased (Fig 3B and 3C). The quantitative real time PCR assay revealed that the expression of *ABCA1* gene was significantly downregulated upon PADK-treatment and that the expression of *NPC1* seem not to be affected (Fig 3D). Significantly lower ABCA1 mRNA expression was also detected under the U18666A- and Leu/NH_4_Cl-treatment (Fig 3D). The *NPC1* gene showed increased expression only in Leu/NH_4_Cl-treated SH-SY5Y cells compared to non-treated cell line.

**Fig 4:**
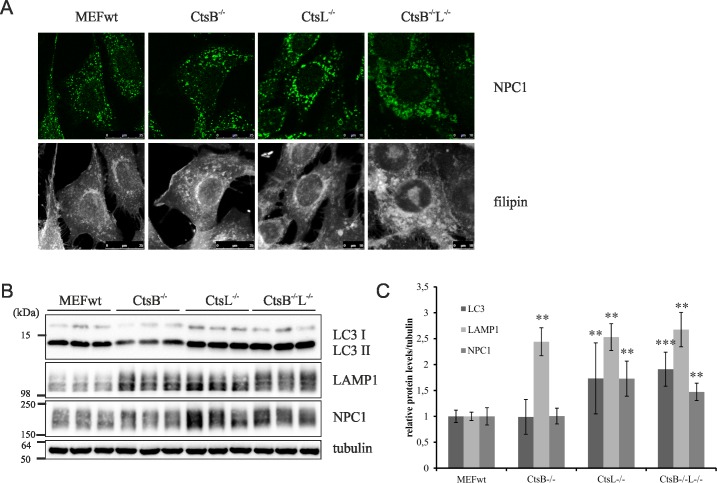
Cathepsin B/L genetic depletion in MEF cells causes accumulation of cholesterol and lysosomal proteins. (A) Confocal microscopy of CtsB KO, CtsL KO and CtsB/L double KO MEFs. Cholesterol (filipin staining, white) and NPC1 (green). (B) Western blot analysis of CtsB KO, CtsL KO and CtsB/L double KO MEFs using LC3, LAMP1 and NPC1 antibody. α-Tubulin was used as a loading control. (C) Quantification of Western blot results of the 3 independent experiments was performed by ImageJ. Student t-test was used for statistical analysis. Error bars present the mean ± standard deviation (** p < 0.01, *** p < 0.001).

**Fig 5:**
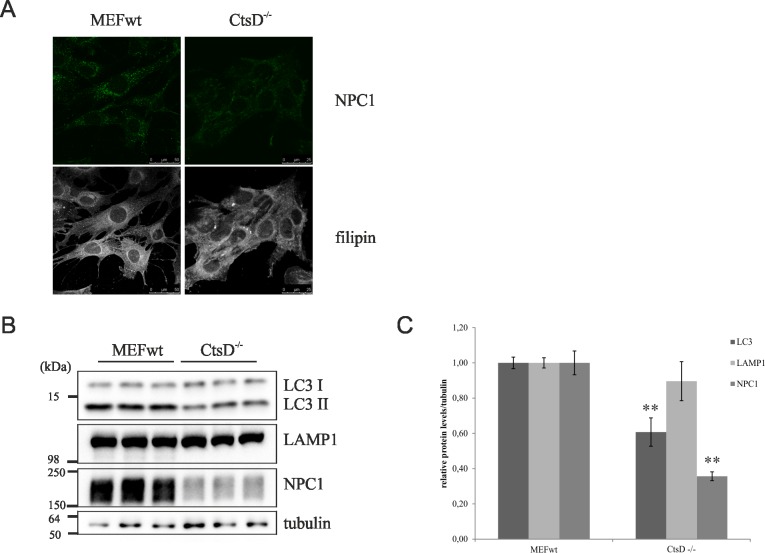
Cathepsin D genetic depletion in MEF cells does not cause cholesterol nor lysosomal proteins accumulation. (A) Confocal microscopy of CtsD KO MEFs. Cholesterol (filipin staining, white) and NPC1 (green). (B) Western blot analysis of CtsD KO MEFs using LC3, LAMP1 and NPC1 antibody. α-Tubulin was used as a loading control. (C) Quantification of Western blot results of the 3 independent experiments was performed by ImageJ. Student t-test was used for statistical analysis. Error bars present the mean ± standard deviation (** p < 0.01).

To confirm the role of CtsB/L in cholesterol transport, and not of CtsD, we used MEF cells which were null for CtsB, CtsL or CtsB and CtsL, and MEFs null for only CtsD, respectively. Indeed, filipin staining of free cholesterol confirmed intracellular cholesterol accumulation in CtsB/L double KO MEFs, while single KO CtsB or CtsL MEFs did not show altered intracellular cholesterol accumulation (Fig 4A). This result suggested that CtsB and CtsL have overlapping roles in controlling intracellular cholesterol transport and that loss of function of both of these cysteine cathepsins causes cholesterol trafficking defect. In addition, cholesterol accumulation in CtsB/L double KO MEFs was accompanied with enlarged punctate NPC1 immunostaining (Fig 4A) similar to that upon CtsB/L inhibition (Fig 2A). Results of western blotting showed that genetic deletion of CtsB/L or CtsL alone causes significant increase of the lysosomal proteins LC3II, LAMP1 and NPC1, and that deletion of CtsB alone causes accumulation of only LAMP1 (Fig 4B and 4C). In contrast to CtsB/L KO MEFs, genetic depletion of CtsD did not cause intracellular cholesterol accumulation nor accumulation of LC3II, LAMP1 and NPC1 (Fig 5). Indeed, in accord to our results of CtsD-inhibition (PepA-treatment) in CHOwt cells (Fig 1D and 1E, and Fig 2), CtsD KO MEFs showed significantly decreased levels of NPC1 (Fig 5A, 5B and 5C) and LC3II (Fig 5B and 5C).

### Cathepsin(s) B/L are essential in lysosomal degradation of the key Alzheimer's proteins

**Fig 6:**
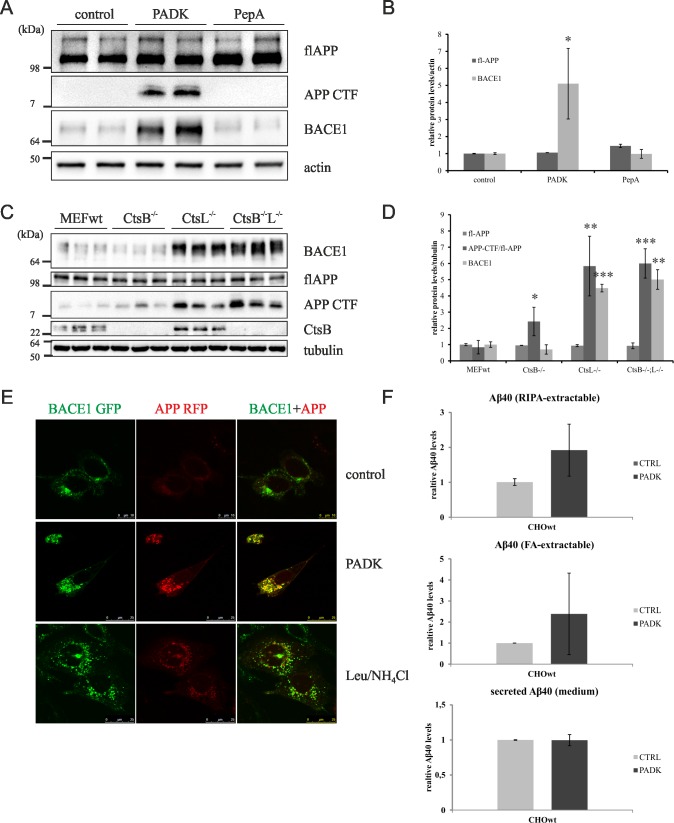
Cathepsin B/L are essential in lysosomal degradation of the key Alzheimer’s proteins. (A) Western blot of CHOwt cells treated with CtsB/L (PADK) and CtsD (PepA) inhibitors. Fl-APP and BACE1 were monitored in cell lysate, APP-CTFs were immunoprecipitated form the cell lysate. β–Actin was used as a loading control. (B) Quantification of Western blot results of the 3 independent experiments was performed by ImageJ. Student t-test was used for statistical analysis. Error bars present the mean ± standard deviation (* p < 0.05). Note: APP-CTF levels in control and PepA treated cells were below ImageJ quantification sensitivity. (C) Western blot analysis of CtsB KO, CtsL KO and CtsB/L double KO MEFs. Fl-APP and BACE1 were monitored in cell lysate, APP-CTFs were immunoprecipitated from the cell lysate. α–Tubulin was used as a loading control. (B) Quantification of Western blot results of the 3 independent experiments was performed by ImageJ. Student t-test was used for statistical analysis. Error bars present the mean ± standard deviation (* p < 0.05, ** p < 0.01, *** p < 0.001). (E) Confocal microscopy of CHOwt cells transiently transfected with BACE1-GFP and APP-RFP plasmids upon CtsB/L inhibition (PADK) and lysosomal inhibition (Leu/NH_4_Cl). (F) ELISA analysis of soluble (RIPA-extractable), insoluble (FA-extractable) and secreted Aβ. Error bars present the mean ± standard deviation.

Genetic variants in CtsD have been identified as a risk factor for Alzheimer's disease [21] and increased immunostaining of CtsD has been reported in AD brains [22]. However, recent study on α-synuclein (α-syn), that accumulates in Parkinson's disease brains, point to specific role of cysteine CtsB and L on α-syn lysosomal degradation [23]. To assess the role of CtsD and/or CtsB/L on degradation of the key Alzheimer's proteins, APP metabolites (Aβ, APP-CTFs, fl-APP) and BACE1, we monitored their levels upon CtsB/L or CtsD inhibition or genetic depletion. Here, we provide evidence that blocking the activity of CtsB/L (PADK) and not CtsD (PepA) in CHOwt cells causes significant accumulation of BACE1 and APP-CTFs (Fig 6A and 6B). Similar increase of these key AD proteins was observed upon PADK-treatment of SH-SY5Y cells (S2 Fig). Using CtsB/L KO MEFs we further confirmed this. In CtsB and L double KO MEFs there is substantial accumulation of BACE1 and APP-CTFs, while the levels of fl-APP seem not to be affected by lysosomal dysfunction due to CtsB/L depletion (Fig 6C and 6D). Single KO CtsB or CtsL MEFs revealed that CtsL may be the major cysteine cathepsin involved in BACE1/APP-CTF lysosomal degradation. In accord to our results on CtsD-inhibitor treated CHOwt cells (Fig 6A), CtsD KO MEFs did not show accumulation of BACE1 (results not shown). Accumulation of APP-CTFs and BACE1 upon CtsB/L inhibition was further investigated by immunofluorescent confocal microscopy which showed increased colocalization and sequestration of both APP and BACE1 in PADK treated CHOwt cells (Fig 6E). We have previously reported similar accumulation of exogenously expressed APP-RFP and BACE1-GFP within endo-lysosomal pathway in *NPC1*-geneticaly deleted CHO cells (*NPC1*-null) [24]. Disturbed trafficking of both APP and BACE1 upon PADK-treatment of CHOwt cells was detected on their endogenous levels as well (S3 Fig). As we have previously shown that sequestration of APP and BACE1 within the endolysosomal system may likely cause increased intracellular Aβ generation in CHO *NPC1*-null cells we further analyzed intracellular and secreted Aβ levels upon PADK-treatment of CHOwt cells. Although we observed an increase in intracellular Aβ levels in PADK-treated CHOwt cells, these changes were not significant probably due to intracellular Aβ variations (Fig 6F). This result, together with our findings on lysosomal impairment and altered intracellular cholesterol trafficking, support that CtsB/L and NPC1 dysfunction may share several features in common.

## Discussion

Lysosomal proteostasis is crucial for maintaining normal lysosomal and cellular functions. Neurons are particularly sensitive to dysfunction of lysosomes as they cannot dilute accumulated toxic molecules and aggregates by cell division. Thus, impairment of lysosomal function plays an important role in neuronal degeneration and in the pathogenesis of number of neurodegenerative diseases. Keeping the lysosomes functional is important for both preventing and treating these devastating disorders. In our previous work we have investigated molecular details of an AD-like phenotype in a rare childhood lysosomal storage disorder NPC, showing that intracellular cholesterol accumulation within the late endosomes/lysosomes causes sequestration of the key AD proteins, APP and BACE1, within endocytic compartments leading to increased intracellular Aβ levels and APP-CTFs [20; 24]. Here, we provide evidence that cysteine cathepsins B/L, and not the aspartyl cathepsin D, are essential in controlling lysosomal function, intracellular cholesterol trafficking and AD-like amyloidogenic features. Our findings implicate that enhancing the activity or levels of CtsB and/or CtsL could provide a promising and a common strategy for maintaining lysosomal function, clearance of the aggregation prone proteins and for preventing and/or treating neurodegenerative diseases.

Firstly, we show that inhibition of CtsB/L in CHOwt and in human neuroblastoma SH-SY5Y cells causes substantial lysosomal dysfunction. This finding was confirmed by several analyses: by monitoring degradation of the EGFR receptor, by labelling of the acidic organelles using LysoTracker dye, immunostaining of NPC1 and by monitoring the levels of several lysosomal proteins such as NPC1, NPC2 and LC3II by Western blotting. Collectively, these studies revealed a significant reduction of the degradation capacity of lysosomes and substantially enlarged and increased lysosomal number upon CtsB/L inhibition. Surprisingly, CtsD inhibition did not cause lysosomal impairment compared to inhibition of CtsB/L implying that cysteine cathepsins CtsB/L represent major lysosomal protease(s). Our findings are in agreement with the recent report which showed that CtsB/L inhibition and not the inhibition of CtsD causes lysosomal dysfunction and consequent cell death in pancreatic beta-cells [25]. Using single and double knockout CtsB and/or CtsL MEFs we revealed that the loss of both CtsB and CtsL acts additively on lysosomal impairment.

Secondly, our results of LysoTracker staining showing similar lysosomal impairment between CHOwt cells treated with the CtsB/L inhibitor and the cells in which cholesterol transporter *NPC1* has been deleted prompted us to speculate that these proteins may play a role in a common cellular pathway and/or that their functions may be interconnected. Indeed, we detected free cholesterol accumulation, a characteristic feature of NPC1 dysfunction in a rare childhood neurodegenerative disorder NPC disease, in both CtsB/L inhibitor treated cells and in CHO *NPC1*-null cells. It has been recently shown that aspartyl cathepsin CtsD most likely indirectly binds to NPC1 and that NPC1 could modulate CtsD levels and processing [26]. Our results of CtsD inhibition and CtsD genetic depletion did not reveal NPC1 mediated intracellular cholesterol accumulation, implying that CtsD protease, in contrast to CtsB/L, may not play a role in intracellular cholesterol transport. Although CtsB has also been detected as a NPC1 binding partner, this has not been further investigated. However, it has been recently shown that secretion of NPC2, an intracellular cholesterol transporter that likely acts in concert with NPC1 to exert cholesterol out of the late endosomes/lysosomes, is reduced upon CtsB/L inhibition while its intracellular levels accumulated [15]. Here, we show that CtsB/L inhibition in CHO and in human neuroblastoma SH-SY5Y cells as well as CtsB and CtsL genetic depletion also affects NPC1 and/or NPC2 and causes their sequestration and accumulation of intracellular free cholesterol. It is not clear how the accumulated NPC1/NPC2 are malfunctional in cholesterol egress and whether the observed effect is primary or the secondary effect of CtsB/L inhibition. Nevertheless, our findings are in accord with the recent study by Hannaford et al. [15] supporting the functional link between cysteine cathepsins B/L and NPC1/NPC2. It is intriguing that NPC1 has shown indispensable for Ebola virus internalization and that in this process CtsB/L are also involved. Thus, it is reasonable to speculate that CtsB/L may regulate NPC1/2 function and cholesterol metabolism. We and others have also shown that CtsB/L inhibition may affect expression of ABCA1 –another cholesterol efflux protein like NPC1/2. Using a single or double knockout CtsB and/or CtsL MEFs we confirmed that the loss of function of both CtsB and CtsL causes intracellular cholesterol accumulation similar to NPC1 dysfunction phenotype in NPC disease. Previously observed selective neuronal vulnerability of cerebellar Purkinje neurons in CtsB^-/-^L^-/-^ mice [14], which is also a characteristic feature of NPC disease, further supports that the loss of CtsB/L likely affects additional enzymes/proteins, such as NPC1/2. In addition, it was reported that CtsB/L KO mice show progressive neuronal loss and substantial brain atrophy [14], similar as NPC1 deficient mice [27;28].

Thirdly, we show that CtsB/L are involved in degradation of the key AD proteins: C-terminal fragments of APP, which are also neurotoxic [29], like Aβ that accumulates in AD brains, and BACE1. BACE1, that codes for β-secretase, an enzyme that performs the first cleavage step towards Aβ generation, has recently become a prime therapeutic target for lowering Aβ and treating AD. As BACE1 has shown to accumulate with ageing [30] and in AD brains [31] reducing its levels and/or activity by its CtsB/L-mediated degradation might be protective against AD. Indeed, cysteine cathepsins CtsB/L have recently been identified as being essential in lysosomal degradation of α-synuclein (α-syn) that accumulates in Parkinson's disease brains [23], further supporting their important role in lysosomal proteostasis and degradation of aggregation prone proteins that accumulate in the neurodegenerative disease brains.

Collectively, this work shows a pivotal role of cysteine cathepsins B and L in maintenance of the lysosomal function and, together with NPC1/NPC2, in the regulation of intracellular cholesterol transport. Moreover, we present evidence that CtsB/L are involved in degradation of the toxic APP metabolites (APP-CTFs and Aβ) as well as BACE1 –the key AD proteins. Their recently identified role in lysosomal degradation of α-syn implies that CtsB/L activation may present a common approach to maintain lysosomal function and to prevent/ameliorate neurodegenerative diseases. Therefore, mechanistic studies to provide an understanding of the CtsB/L-mediated regulation of lysosomal function, cholesterol transport and degradation of aggregated proteins are needed to understand the pathogenesis of many diseases.

## Supporting Information

S1 Fig Rapamycin treatment further increases LC3II/actin ratio in PADK- and Leu/NH_4_Cl-treated CHO cells. (A) Western blot of LC3 in untreated and rapamycin-treated (rapamycin, Sigma-Aldrich (5μg/ml/24h)) CHOwt cells, PADK- and Leu/NH_4_Cl-treated cells. β-Actin was used as a loading control. (B) Quantification of Western blot results of the 3 independent experiments was performed by ImageJ. Student t-test was used for statistical analysis. Error bars present the mean ± standard deviation (* p < 0.05).
(TIF)

S2 Fig PADK treatment of SH-SY5Y cells causes impairment in lysosomal degradation of the key Alzheimer’s proteins. (A) Western bolt of SH-SY5Y cells treated with PADK. Fl-APP and BACE1 were monitored in cell lysates, APP-CTFs were immunoprecipitated from the cell lysates. α-Tubulin was used as a loading control. (B) Quantification of Western blot results of the 3 independent experiments was performed by ImageJ. Student t-test was used for statistical analysis. Error bars present the mean ± standard deviation (** p < 0.01, *** p < 0.001).
(TIF)

S3 Fig Endogenous APP and BACE1 show altered localization in PADK-treated CHOwt cells. Confocal microscopy of control and PADK treated CHOwt cells. APP (red), BACE1 (green). Immunostaining of APP was performed using APP C terminal antibody C1/6.1 (1:100, kindly provided by R. Nixon), while BACE1 was stained using an anti-BACE1 D10E5 (1:100, Cell Signaling).
(TIF)
