# Supplementary figures and images for: Loss of Cathepsin B and L Leads to Lysosomal Dysfunction, NPC-Like Cholesterol Sequestration and Accumulation of the Key Alzheimer's Proteins
Source: PLoS One. 2016 Nov 30;11(11):e0167428. doi: 10.1371/journal.pone.0167428 (PMC5130271; doi:10.1371/journal.pone.0167428)

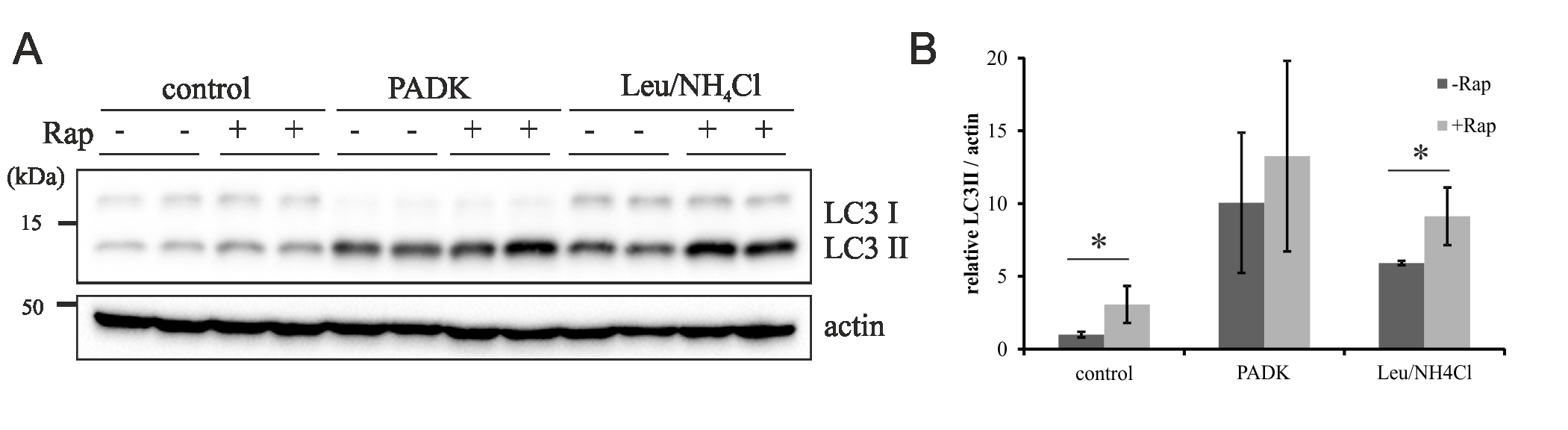

Supplement: S1 Fig — (A) Western blot of LC3 in untreated and rapamycin-treated (rapamycin, Sigma-Aldrich (5μg/ml/24h)) CHOwt cells, PADK- and Leu/NH4Cl-treated cells. β-Actin was used as a loading control. (B) Quantification of Western blot results of the 3 independent experiments was performed by ImageJ. Student t-test was used for statistical analysis. Error bars present the mean ± standard deviation (* p < 0.05). (TIF) [file pone.0167428.s001.tif]

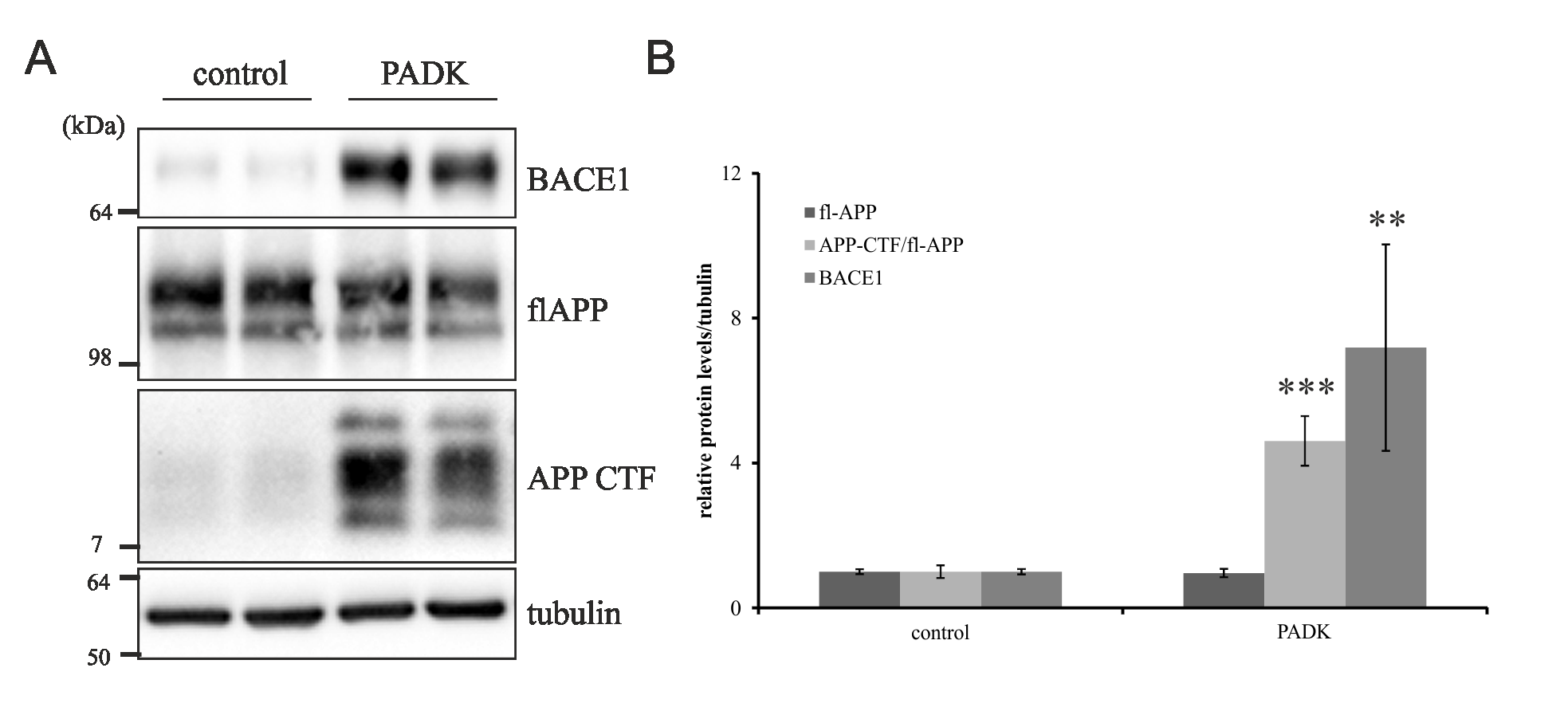

Supplement: S2 Fig — (A) Western bolt of SH-SY5Y cells treated with PADK. Fl-APP and BACE1 were monitored in cell lysates, APP-CTFs were immunoprecipitated from the cell lysates. α-Tubulin was used as a loading control. (B) Quantification of Western blot results of the 3 independent experiments was performed by ImageJ. Student t-test was used for statistical analysis. Error bars present the mean ± standard deviation (** p < 0.01, *** p < 0.001). (TIF) [file pone.0167428.s002.tif]

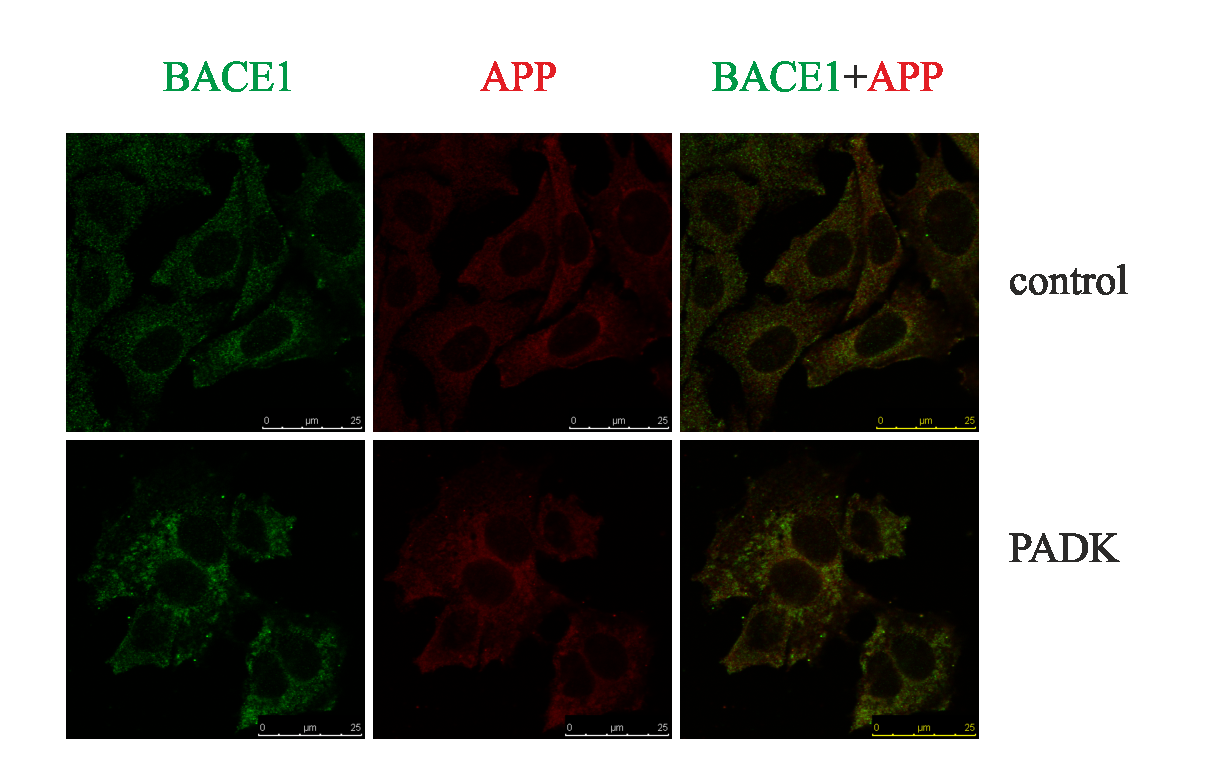

Supplement: S3 Fig — Confocal microscopy of control and PADK treated CHOwt cells. APP (red), BACE1 (green). Immunostaining of APP was performed using APP C terminal antibody C1/6.1 (1:100, kindly provided by R. Nixon), while BACE1 was stained using an anti-BACE1 D10E5 (1:100, Cell Signaling). (TIF) [file pone.0167428.s003.tif]
